# Low cholesterol level associated with severity and outcome of spontaneous intracerebral hemorrhage: Results from Taiwan Stroke Registry

**DOI:** 10.1371/journal.pone.0171379

**Published:** 2017-04-19

**Authors:** Yu-Wei Chen, Chen-Hua Li, Chih-Dong Yang, Chung-Hsiang Liu, Chih-Hung Chen, Jau-Jiuan Sheu, Shinn-Kuang Lin, An-Chih Chen, Ping-Kun Chen, Po-Lin Chen, Chung-Hsin Yeh, Jiunn-Rong Chen, Yu-Jen Hsiao, Ching-Huang Lin, Shih-Pin Hsu, Tsang-Shan Chen, Sheng-Feng Sung, Shih-Chieh Yu, Chih-Hsin Muo, Chi Pang Wen, Fung-Chang Sung, Jiann-Shing Jeng, Chung Y. Hsu, Taiwan Stroke Registry Investigators

**Affiliations:** 1 Department of Neurology, Taiwan Landseed Hospital, Taoyuan, Taiwan; 2 Department of Neurology, National Taiwan University Hospital, Taipei, Taiwan; 3 Department of Neurosurgery, Taiwan Landseed Hospital, Taoyuan, Taiwan; 4 Department of Neurology, China Medical University Hospital, Taichung, Taiwan; 5 Department of Neurology, National Cheng Kung University Hospital, College of Medicine, National Cheng Kung University, Tainan, Taiwan; 6 Department of Neurology, Taipei Medical University Hospital, Taipei, Taiwan; 7 Department of Neurology, Taipei Tzu Chi Hospital, and School of Medicine, Tzu Chi University, Hualien, Taiwan; 8 Department of Neurology, Chung Shan Medical University Hospital, Taichung, Taiwan; 9 Department of Neurology, Lin Shin Hospital, Taichung, Taiwan; 10 Department of Neurology, Taichung Veterans General Hospital, Taichung, Taiwan; 11 Department of Neurology, Yuan Rung Hospital, Changhua, Taiwan; 12 Department of Sport and Health Management, Da-Yeh University, Changhua, Taiwan; 13 Department of Nursing, College of Medicine & Nursing, Hung-Kuang University, Taichung, Taiwan; 14 Department of Neurology, Yunlin Christian Hospital, Yunlin, Taiwan; 15 Department of Neurology, National Taiwan University Hospital—Yunlin Branch, Yunlin, Taiwan; 16 Department of Neurology, Kaohsiung Veterans General Hospital, Kaohsiung, Taiwan; 17 Department of Neurology, E-Da Hospital, Kaohsiung, Taiwan; 18 Department of Neurology, Sin-Lau Hospital, Tainan, Taiwan; 19 Division of Neurology, Department of Internal Medicine, Ditmanson Medical Foundation Chia-Yi Christian Hospital, Chiayi, Taiwan; 20 Department of Neurology, Kuang Tien General Hospital, Taichung, Taiwan; 21 Management Office for Health Data, China Medical University Hospital, Taichung, Taiwan; 22 National Health Research Institutes, Miaoli, Taiwan; 23 Department of Health Services Administration, China Medical University, Taichung, Taiwan; 24 Stroke Center, National Taiwan University Hospital, Taipei, Taiwan; 25 Graduate Institute of Clinical Medical Science, China Medical University, Taichung, Taiwan; University of British Columbia, CANADA

## Abstract

The relationship between cholesterol level and hemorrhagic stroke is inconclusive. We hypothesized that low cholesterol levels may have association with intracerebral hemorrhage (ICH) severity at admission and 3-month outcomes. This study used data obtained from a multi-center stroke registry program in Taiwan. We categorized acute spontaneous ICH patients, based on their baseline levels of total cholesterol (TC) measured at admission, into 3 groups with <160, 160–200 and >200 mg/dL of TC. We evaluated risk of having initial stroke severity, with National Institutes of Health Stroke Scale (NIHSS) >15 and unfavorable outcomes (modified Rankin Scale [mRS] score >2, 3-month mortality) after ICH by the TC group. A total of 2444 ICH patients (mean age 62.5±14.2 years; 64.2% men) were included in this study and 854 (34.9%) of them had baseline TC <160 mg/dL. Patients with TC <160 mg/dL presented more often severe neurological deficit (NIHSS >15), with an adjusted odds ratio [aOR] of 1.80; 95% confidence interval [CI], 1.41–2.30), and 3-month mRS >2 (aOR, 1.41; 95% CI, 1.11–1.78) using patients with TC >200 mg/dL as reference. Those with TC >160 mg/dL and body mass index (BMI) <22 kg/m^2^ had higher risk of 3-month mortality (aOR 3.94, 95% CI 1.76–8.80). Prior use of lipid-lowering drugs (2.8% of the ICH population) was not associated with initial severity and 3-month outcomes. A total cholesterol level lower than 160 mg/dL was common in patients with acute ICH and was associated with greater neurological severity on presentation and poor 3-month outcomes, especially with lower BMI.

## Introduction

Hypercholesterolemia is associated with increased risks of coronary artery events, coronary revascularization and ischemic stroke. Reduction of low-density lipoprotein cholesterol (LDL-C) with lipid-lowering agents has been demonstrated to significantly reduce the cardiovascular risks [1]. However, the relationship between cholesterol levels and stroke seems less evident than the relationship between the extent of reduction of LDL-C and cardiovascular events. Previous epidemiologic studies showed that hypercholesterolemia was associated with a lower risk of intracerebral hemorrhage (ICH) [2], while the low LDL-C level increased the risk of ICH mortality [3, 4]. A recent systematic review and meta-analysis reported an inverse relationship between total cholesterol (TC) levels and the risk of hemorrhagic stroke [5]. However, there were conflicting study findings on the association between lipid-lowering medications and the risk of ICH [6, 7]. A meta-analysis of randomized controlled trials showed that statins, the medication of choice for hypercholesteremia, may reduce the overall incidence of stroke [8], but conflicting results were also found in the impact of prior use of statins on the prognosis of ICH; it could be neutral [4], favorable with reduced mortality [9], or even worse [10]. The relationship between TC and ICH risk has not been well studied yet for the Asian population, which may be different from Western populations. Therefore, this study used a multi-center stroke registry to investigate the relationship between serum cholesterol level and the severity and prognosis of acute ICH, and functional outcome and deaths at 3 month after stroke.

## Methods

### Patients

The Taiwan Stroke Registry (TSR) is a nationwide prospective registry with 39 participating stroke centers. The details of diagnosis, inclusion criteria and collection of variables of this program has been presented elsewhere [11]. In brief, patients with an acute cerebrovascular event were prospectively enrolled in the registry within 10 days of admission or during hospitalization and followed up prospectively. An expert panel established a consensus protocol on data collection criteria, including demographic data, related medications, etiological factors, clinical course, prognosis, complications of the index cerebrovascular events. Diagnosis and stroke subtypes were determined based on clinical features and laboratory examinations, brain imaging, echocardiography, vascular ultrasonography and angiography. All patients with non-traumatic ICH were referred to neurologists or neurosurgeons for confirmation of the diagnosis. Patients with ICH secondary to acute cerebral infarct were excluded from the data analysis. Stroke patients were scheduled to follow-up checkups at 1, 3, 6 and 12 months after stroke onset. The Taiwan Stroke Registry was approved by “Research Ethics Committee, China Medical University and Hospital, Taichung, Taiwan” and every Institutional Review Board of each stroke center approved the participation in the registry program. Written informed consent for participation in the registry program was obtained from each patient or his/her legal representatives with the approval of attending physician. A numeric code was assigned to each patient whose clinical data in the registry containing no individual identification numbers or any privacy data. The whole data set, a big database, has been approved for big data analytics for research topics such as the one in this study.

More than 40,000 stroke patients were registered from 1st May 2006 to 30^th^ April 2009. Patients without data of fasting TC during hospitalization were excluded from this study. Patients were categorized into three groups based on the baseline levels: <160, 160–200, and >200 mg/dL of TC. The cut-off points of 160 and 200 mg/dL were adopted from the Framingham Heart Study [12] and Adult Treatment Panel (ATP) III classification [13]. Both National Institute of Health Stroke Scale (NIHSS) and Glasgow Coma Scale (GCS) were recorded at the emergent room or at the admission to assess the initial severity of stroke. The baseline scores of NIHSS were stratified by the cut points of 14 and 24 [14] and GCS were stratified by the cut points of 4 and 12. Patients with a NIHSS >15 were defined as severe and very severe stroke cases [15]. Modified Rankin Scales (mRS) and deaths identified within 3 months after stroke were used to assess functional outcomes and the risk of mortality. The vascular risk factors were defined to conform to the consensus of TSR criteria [11]. The functional outcome and vital status of all patients were determined by examining medical records and/or telephone interviews.

### Statistical analysis

Data analysis first compared distributions of sex, mean age, mean body mass index, risk factors of stroke, medication history and laboratory data among three TC groups (levels of <160, 160–200 and >200 mg/dL). We used χ^2^ test to examine the distribution of each categorical variable among these TC groups and used Kruskall-Wallis test to examine differences among means, using a significance level of 0.05. Because the baseline characteristics of ICH patients in three groups may confound the outcome of interests, we further selected the study subjects with the propensity score matching method to determine if there was significant difference of clinical characteristics among the three groups.

We stratified TC into 11 levels to determine the relationship between mean initial NIHSS scores and TC, which was in a 20 mg/dL increment of TC, from <100, 100–119, and through ≥280 mg/dL. Univariate and multivariate logistic regression analyses were used to determine crude odds ratios (OR) and adjusted odds ratios (aOR), respectively, and 95% confidence intervals (CI) of initial NIHSS score >15, of 3-month mRS >2 and of 3-month mortality in association with TC using patients with TC >200 mg/dL as the reference group. Further data analysis evaluated the joint effect of TC (<160 vs. ≥160 mg/dL) and body mass index (<22.0, 22.0–26.9 and ≥27.0 kg/m^2^) on the outcomes of interest. The multivariate analysis first included only sex and age for adjustment. Further analysis included variables significant at *p* <0.05 in the univariate analysis and variables with biological plausibility. We used the SAS statistical package (version 9.1; SAS Institute, Cary, NC, USA) to perform data analysis.

## Results

**Fig 1:**
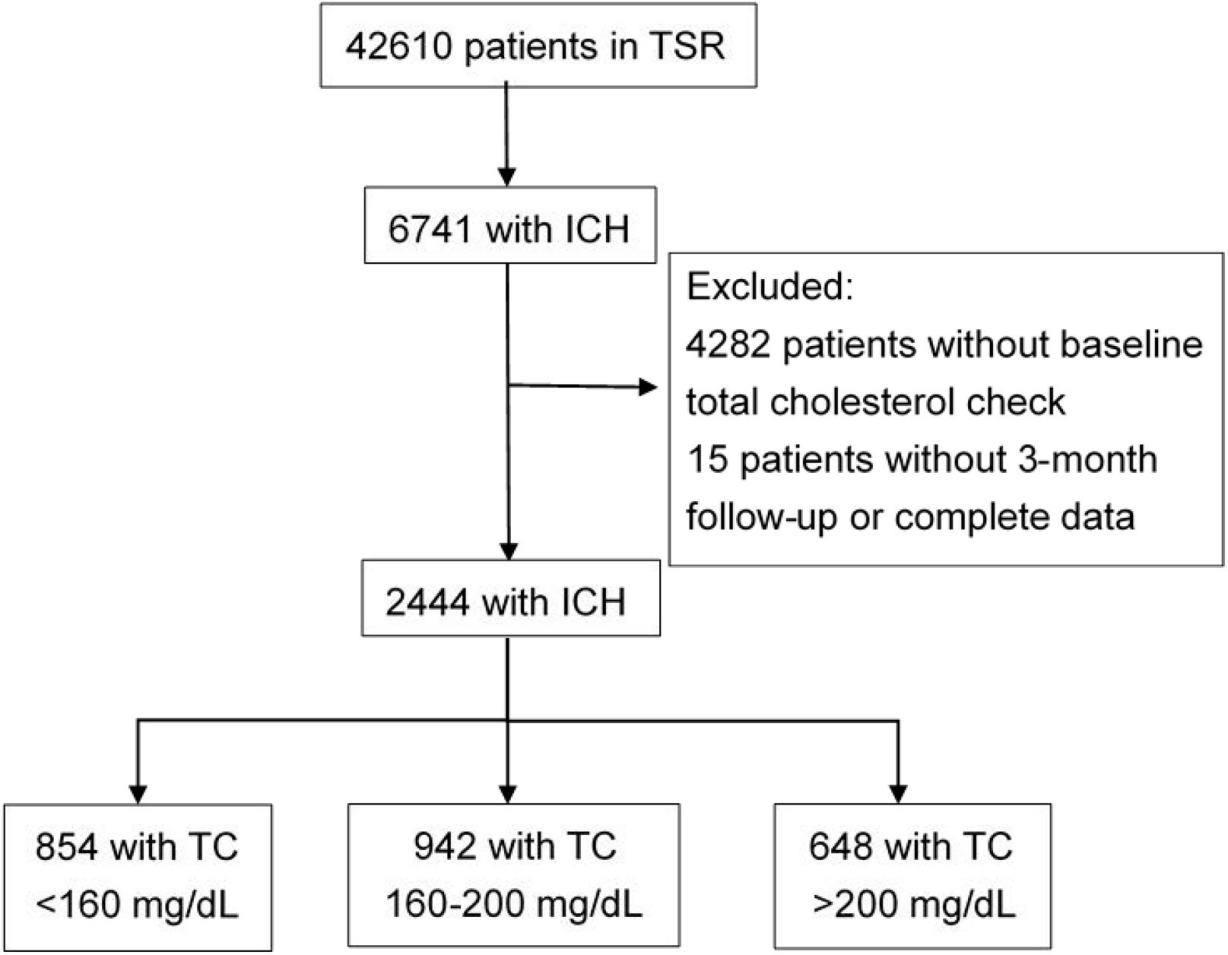
Flowchart for selection of spontaneous ICH patients in the Taiwan Stroke Registry. ICH: intracerebral hemorrhage; TC: total cholesterol levels.

A total of 2444 spontaneous ICH patients with baseline fasting TC levels measured immediately after stroke were included in this study after excluding 4139 patients without fasting TC records from the TSR (Fig 1). Of these patients (mean age, 62.5±14.2 years; men, 64.2%), 13.7% had previous ischemic stroke or transient ischemic attack and 7.8% had a history of ICH (Table 1). The average time from symptom onset to hospital arrival was 5.3±7.8 hours. Patients on admission presented with a median GCS score of 15 (interquartile range [IQR], 11–15) and a median NIHSS score of 9 (IQR, 4–18). The site of ICH occurred most frequently at the putamen (31.6%), followed by the thalamus (19.2%), lobes (12.8%), pons (6.8%), cerebellum (6.7%), and intraventricular hemorrhage (2.2%). Sixteen percent of patients had ICH at more than one site. During hospitalization, 14.6% of them had received hematoma evacuation or ventricular drainage surgery. The overall case-fatality rate of ICH at discharge was 4.9% and at 3-month was 8.2%. The median mRS at discharge was 4 (IQR, 2–5), with 48.8% of patients had a mRS of >2.

**Table 1:** Characteristics of patients with intracerebral hemorrhage by the levels of initial total cholesterol.

		Total cholesterol levels			
	All patients (n = 2444)	< 160 mg/dL (n = 854)	160–200 mg/dL (n = 942)	> 200 mg/dL (n = 648)	*P* value
Age, y	62.5±14.2	65.0±14.8	62.3±14.2	59.5±12.9	<0.0001
Male	1570 (64.2)	618 (72.4)	593 (63.0)	359 (55.4)	<0.0001
Body mass index, kg/m^2^	24.7±4.3	23.9±4.3	24.8±4.3	25.5±4.3	<0.0001
Medical history					
Hypertension	2102 (86.6)	715 (84.2)	809 (86.4)	578 (89.9)	0.006
Diabetes mellitus	619 (25.6)	222 (26.3)	227 (24.3)	170 (26.4)	0.55
Previous ischemic stroke / TIA	334 (13.7)	137 (16.1)	117 (12.5)	80 (12.4)	0.04
Previous ICH	187 (7.8)	74 (8.8)	71 (7.7)	42 (6.6)	0.28
Atrial fibrillation	116 (4.8)	65 (7.6)	33 (3.5)	18 (2.8)	<0.0001
ESRD	84 (3.5)	43 (5.1)	25 (2.7)	16 (2.5)	0.007
Smoking	898 (37.1)	335 (39.7)	347 (37.1)	216 (33.5)	0.049
Alcohol consumption	525 (21.6)	190 (22.5)	198 (21.1)	137 (21.3)	0.77
Pre-ICH medications					
Lipid-lowering drugs	68 (2.8)	25 (2.9)	22 (2.3)	21 (3.2)	0.53
Antiplatelets	200 (8.2)	84 (9.8)	69 (7.3)	47 (7.3)	0.09
Anticoagulants	43 (1.8)	18 (2.1)	17 (1.8)	8 (1.2)	0.44
Anti-hypertensive drugs	971 (39.7)	347 (40.6)	373 (39.6)	251 (38.7)	0.75
Anti-diabetic drugs	321 (13.1)	123 (14.4)	111 (11.8)	87 (13.4)	0.25
Laboratory data^†^					
Systolic blood pressure, mmHg	178.2±36.2	174.6±37.4	179.5±35.5	181.2±35.3	0.0009
Diastolic blood pressure, mmHg	100.8±23.3	97.5±23.7	102.0±22.3	103.4±23.7	<0.0001
Hematocrit, %	40.8±5.9	39.5±6.3	41.4±5.2	41.8±5.8	<0.0001
White blood cell count, cumm	9.5±9.7	9.4±6.7	9.5±13.9	9.50±3.7	0.96
Platelet count, cumm	209.1±79.6	195.9±77.2	212.6±82.3	221.2±76.3	<0.0001
Creatinine, mg/dL	1.4±1.6	1.5±1.8	1.3±1.4	1.3±1.6	0.02
Sugar, mg/dL	152.3±68.8	151.8±63.4	150.8±68.3	156.5±76.1	0.24
International normalized ratio	1.3±1.5	1.3±1.6	1.2±1.2	1.3±1.6	0.21
NIHSS score on admission					
0–14	1592 (69.1)	484 (60.1)	658 (73.9)	450 (74.1)	<0.0001
15–24	360 (15.6)	149 (18.5)	127 (14.3)	84 (13.8)	
≥25	352 (15.3)	173 (21.5)	106 (11.9)	73 (12.0)	
Median (IQR)^#^	9 (4–18)	11 (5–23)	8 (4–16)	8 (4–15)	<0.0001
Initial GCS					
3–4	98 (4.1)	52 (6.2)	28 (3.0)	18 (2.8)	<0.0001
5–12	683 (28.4)	292 (35.0)	230 (24.8)	162 (25.2)	
≥13	1621 (67.5)	491 (58.8)	671 (72.2)	459 (71.9)	
Median (IQR)^#^	15 (11–15)	14 (9–15)	15 (12–15)	15 (12–15)	<0.0001
Hematoma enlargement	120 (4.9)	42 (4.9)	51 (5.4)	27 (4.2)	0.53
Surgery for ICH	356 (14.6)	163 (19.1)	113 (12.0)	80 (12.4)	<0.0001
Stroke-in-evolution	92 (3.8)	41 (4.8)	34 (3.6)	17 (2.6)	0.09
Infratentorial ICH	194 (7.9)	59 (6.9)	81 (8.6)	54 (8.3)	0.38
Outcome at 3 months					
Death	200 (8.2)	108 (12.7)	60 (6.4)	32 (4.9)	<0.0001
Modified Rankin Scale >2	1082 (48.8)	430 (55.5)	401 (46.9)	251 (42.8)	<0.0001

Data were presented as mean ± standard deviation or number (percentage) unless otherwise indicated.

TIA: transient ischemic attack, ICH: intracerebral hemorrhage; ESRD: end stage renal disease; NIHSS: National Institute of Health and Stroke Scale.

Chi-square test

^†^ ANOVA test and

^#^Kruskal-Wallis test.

Table 1 shows that about one third (854, 34.9%) of patients had a baseline fasting TC <160 mg/dL and about one fourth (648, 26.5%) had TC >200 mg/dL. Patients with TC <160 mg/dL were older and mainly male, and had a lower BMI. These patients were also more prevalent with previous ischemic stroke and transient ischemic attack, atrial fibrillation, end-stage renal disease and smoking, but less prevalent with hypertension. There was a positive association between TC levels and blood pressures, hematocrit and platelet counts, but a reverse association between TC levels and creatinine levels. The neurological presentation in Table 1 also shows that patients with TC <160 mg/dL had higher NIHSS and lower GCS scores, were more likely to receive surgery for ICH, and had a higher rate of mRS >2 and a higher 3-month mortality.

**Fig 2:**
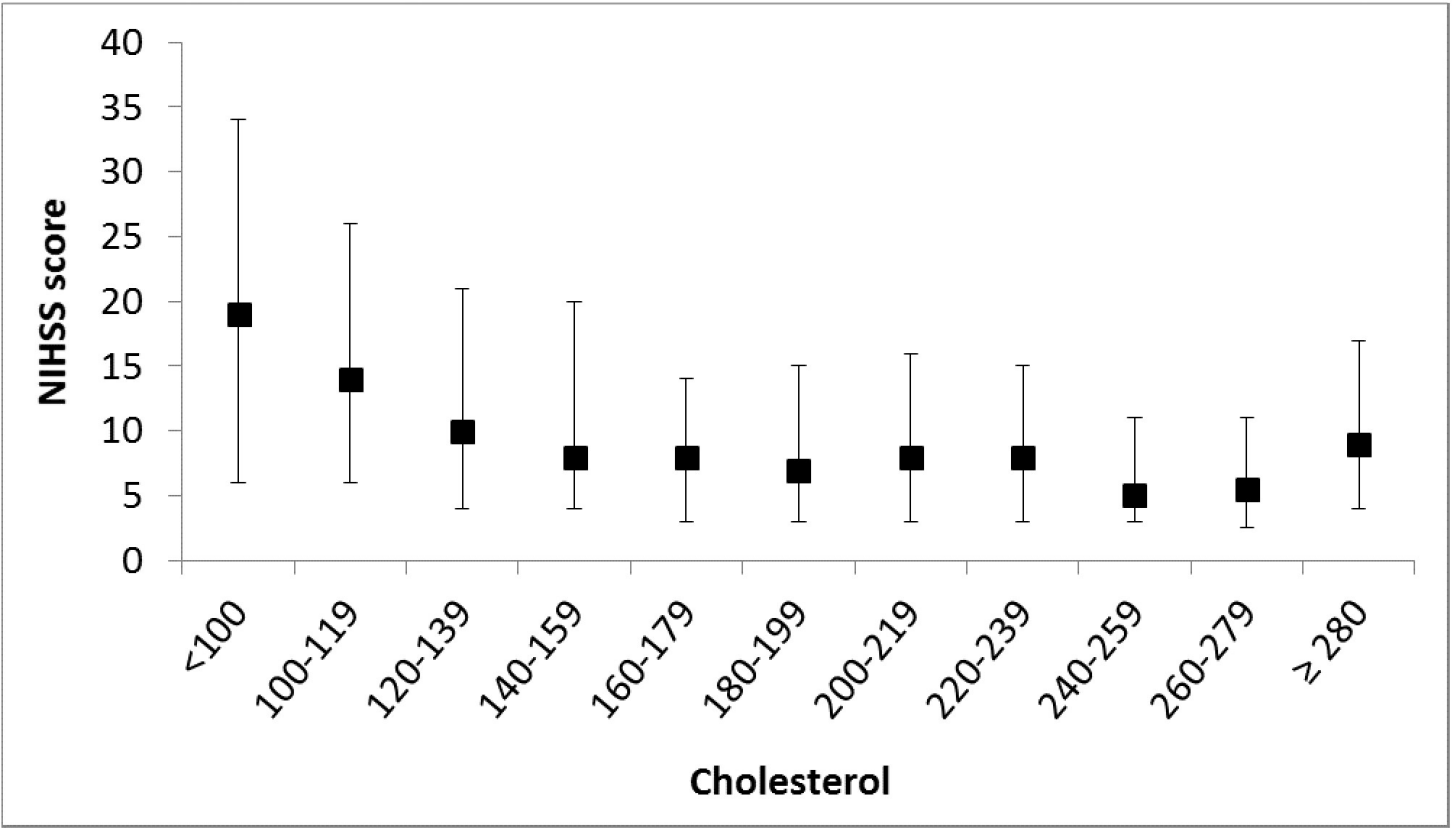
Correlation between initial NIHSS score (median [interquartile range]) and total cholesterol levels in 2444 ICH patients. (Pearson correlation coefficient = -0.74, *p* = 0.009).

**Fig 3:**
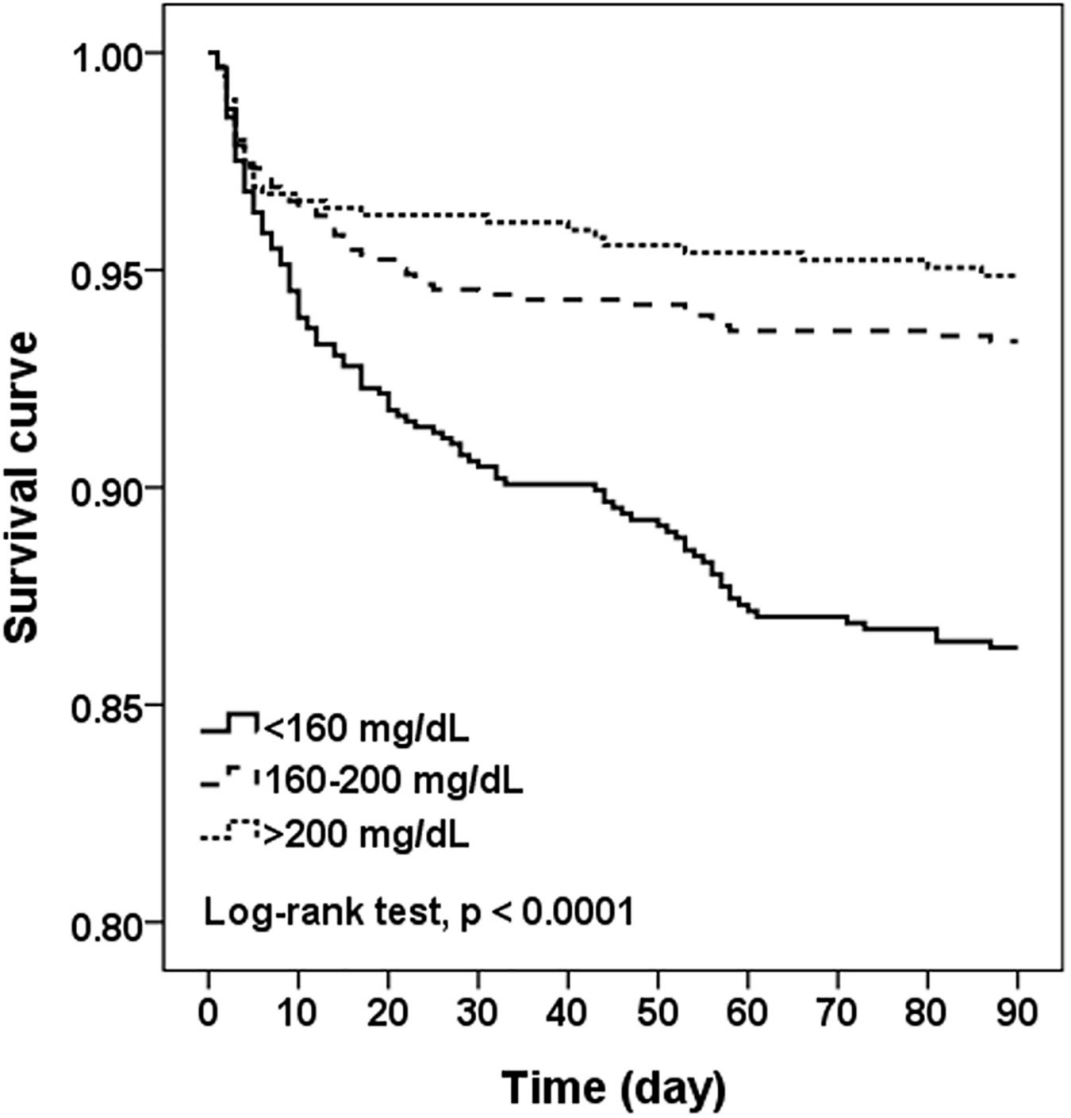
Three-month survival in different cholesterol groups in 2444 ICH patients.

Fig 2 shows that the mean NIHSS score decreased from 20 points for patients with a TC <100 mg/dL to 11 points for those with a TC ≥280 mg/dL (Pearson correlation coefficient = -0.74, *P* = 0.009). Compared to patients with TC >200 mg/dL, those with TC <160 mg/dL had higher frequency of initial NIHSS >15 (aOR, 1.80; 95% CI, 1.41–2.30) (Table 2). The corresponding adjusted ORs of 3-month mRS >2 and 3-month mortality were 1.41 (95% CI, 1.11–1.78) and 2.19 (95% CI, 1.44–3.33). Fig 3 shows that the 3-month survival for patients with TC <160 mg/dL was significantly lower than those with TC 160–200 mg/dL and >200 mg/dL (86.3% vs. 93.4% and 96.9%, *P*<0.001 by log-rank test). By the propensity score matching method, there was still significantly inverse association between initial NIHSS and TC levels, but the association of 3-month outcome and TC levels was borderline.

**Table 2:** Association between initial stroke severity and three month outcome by total cholesterol levels and pre-ICH lipid-lowering drugs use.

	Odds ratio (95% confidence intervals)		
	Crude	Age/sex-adjusted	Multivariate adjusted§
**All ICH patients (n = 2444)**			
***Initial NIHSS score >15***			
Total cholesterol >200 mg/dL	1.00	1.00	1.00
160–200 mg/dL	1.02 (0.80–1.28)	1.01 (0.80–1.29)	1.03 (0.81–1.32)
<160 mg/dL	1.91 (1.52–2.40)‡	1.92 (1.52–2.44)‡	1.80 (1.41–2.30)‡
Pre-ICH lipid-lowering drugs use	0.74 (0.42–1.31)	0.74 (0.42–1.31)	0.67 (0.37–1.21)
***3-month mRS >2***			
Total cholesterol >200 mg/dL	1.00	1.00	1.00
160–200 mg/dL	1.18 (0.95–1.46)	1.09 (0.87–1.35)	1.10 (0.88–1.38)
<160 mg/dL	1.66 (1.34–2.07)‡	1.45 (1.16–1.82)†	1.41 (1.11–1.78)†
Pre-ICH lipid-lowering drugs use	0.72 (0.43–1.21)	0.72 (0.43–1.22)	0.59 (0.34–1.02)
***3-month mortality***			
Total cholesterol >200 mg/dL	1.00	1.00	1.00
160–200 mg/dL	1.30 (0.85–1.99)	1.23 (0.81–1.89)	1.27 (0.81–1.97)
<160 mg/dL	2.69 (1.82–4.00)‡	2.40 (1.60–3.60)‡	2.19 (1.44–3.33)‡
Pre-ICH lipid-lowering drugs use	0.90 (0.37–2.19)	0.92 (0.38–2.23)	0.80 (0.33–1.97)
**Selected ICH patients by Propensity Score Matching Method (n = 747)**			
***Initial NIHSS score >15***			
Total cholesterol >200 mg/dL	1.00	1.00	1.00
160–200 mg/dL	0.97 (0.64–1.47)	0.97 (0.64–1.47)	0.87 (0.56–1.37)
<160 mg/dL	1.62 (1.09–2.40)*	1.62 (1.09–2.40)*	1.70 (1.11–2.60)*
Pre-ICH lipid-lowering drugs use	1.50 (0.65–3.48)	1.49 (0.64–3.48)	1.52 (0.56–4.11)
***3-month mRS >2***			
Total cholesterol >200 mg/dL	1.00	1.00	1.00
160–200 mg/dL	1.28 (0.99–1.84)	1.26 (0.87–1.85)	1.26 (0.84–1.88)
<160 mg/dL	1.28 (1.88–1.84)‡	1.30 (0.89–1.89)	1.37 (0.92–2.04)
Pre-ICH lipid-lowering drugs use	0.81 (0.35–1.87)	0.76 (0.32–1.81)	0.65 (0.24–1.73)
***3-month mortality***			
Total cholesterol >200 mg/dL	1.00	1.00	1.00
160–200 mg/dL	1.97 (0.90–4.34)	1.93 (0.87–4.25)	1.79 (0.74–4.33)
<160 mg/dL	2.09 (0.96–4.56)	2.01 (0.92–4.41)	2.23 (0.94–5.31)
Pre-ICH lipid-lowering drugs use	2.87 (0.94–8.70)	2.90 (0.94–8.93)	1.53 (0.35–6.80)

*, indicating *P*<0.05

†, *P*<0.001

‡, *P*<0.0001.

§, model adjusted for age, gender, all medical history, pre-ICH medications and laboratory data.

Table 3 shows that the estimated risks of NIHSS >15 points were higher for patients with TC<160 mg/dL in any BMI category using TC **>**160 mg/dL and BMI >27.0 kg/m^2^ as the reference. The estimated risks of 3-month mRS >2 and 3-month mortality were higher in patients with TC <160 mg/dL and BMI <22 kg/m^2^ or 22.0–26.9 kg/m^2^. In the groups of TC≥ 160 mg/dL, BMI <22.0 kg/m^2^ had significantly higher risks of initial NIHSS >15, 3-month mRS >2 and mortality, with aORs of 1.81 (95% CI, 1.31–2.52), 1.53 (95% CI, 1.14–2.07) and 3.94 (95% CI, 1.76–8.80), respectively. Patients with both TC <160 mg/dL and BMI <22.0 kg/m^2^ had an aOR of 3-month mortality of 4.11(95% CI, 1.71–9.86). Among the groups with BMI >27.0 kg/m^2^, those with TC <160 mg/dL had a significant higher aOR of initial NIHSS score>15 (2.09; 95% CI 1.33–3.30).

**Table 3:** Odds ratios (95% confidence intervals) of three month outcome by total cholesterol levels and body mass index.

	Total CHO <160 mg/dL			Total CHO >160 mg/dL		
	BMI (kg/m^2^)			BMI (kg/m^2^)		
	<22.0	22.0~26.9	>27.0	<22.0	22.0~26.9	>27.0
Initial NIHSS score >15	3.49 (2.47–4.94)‡	1.55 (1.06–2.25)‡	2.09 (1.33–3.30)*	1.81 (1.31–2.52)*	1.23 (0.89–1.70)	1.00
3-month mRS >2	2.52 (1.80–3.51)‡	1.31 (0.93–1.84)‡	1.51 (0.99–2.31)	1.53 (1.14–2.07) *	1.42 (1.07–1.88)*	1.00
3-month mortality	4.11 (1.71–9.86)*	2.77 (1.12–6.85)*	2.46 (0.89–6.84)	3.94 (1.76–8.80)†	2.64 (1.17–5.96)*	1.00

*, indicating *P* <0.05

†, *P*<0.001

‡, *P*<0.0001.

BMI, body mass index.

Model adjusted for age, gender, hypertension, diabetes mellitus, previous stroke, atrial fibrillation, end-stage renal disease, smoking and pre-ICH lipid-lowering drugs use.

All interaction test *P*>0.05.

## Discussion

The proportion of spontaneous ICH was 16.1% in all stroke patients registered in the TSR [11], similar to those in Western stroke populations (10–15%) [16] and Japanese population (18%) [17], but lower than those in China (24–64%) [18]. Epidemiological studies have shown no obvious declining incidence of ICH in the past decade in spite of improved control of hypertension, which remains a leading cause of ICH [19–21]. It could be partly explained by the aging population worldwide and the increasing use of antithrombotic medications for preventing ischemic stroke and coronary heart diseases [19, 22].

Although epidemiological studies, clinical studies and meta-analysis have shown that higher cholesterol levels are significantly associated with an elevated risk of coronary heart disease [23–25], the relationship between cholesterol and cerebrovascular disease is complex. While there is evidence relating higher cholesterol levels to significantly increased mortality from ischemic stroke, evidence also shows an inverse relationship between TC levels and hemorrhagic stroke risks [5]. Lower cholesterol levels have been associated with increased mortality from intracranial hemorrhage [26, 27], being more prominently in the elderly [24, 26].

In the present study, ICH patients with baseline TC lower than 160 mg/dL had greater initial stroke severity and higher 3-month mortality. Acute hematoma growth of ICH might explain the early neurological deterioration and mortality, especially for those with lower LDL-C levels [28]. Some studies showed that low LDL-C levels were associated with worse initial severity of ICH patients and with higher in-hospital ICH mortality [4, 29]. Among our ICH patients, 7.8% had past history of ICH, which could be a risk factor for recurrent ICH, with annual recurrent rates from 2.1 to 3.2% [30–32]. ICH recurrence is considered as one of the adverse effects of treatment for hyperlipidemia [33].

Is dyslipidemia *per se* or intensive lipid-lower treatment related to greater severity and worse outcome of ICH? Prospective studies supported the association between low LDL-C levels and the ICH risk [3, 34]. Wang et al. used a large meta-analysis to demonstrate the inversed association between lower TC levels and the risk of hemorrhagic stroke [5]. Prospective studies in Japan also showed an elevated risk of ICH mortality in patients with LDL-C <80 mg/dL or TC <160 mg/dL [1, 3]. The interaction of use of statins and TC levels exhibit higher risk towards the ICH [35]. These findings are warning indications that aggressive lipid-lower therapy might be a concern on the ICH risk. Low cholesterol levels resulting from treating patients with cardiovascular diseases and ischemic stroke may increase the ICH risk instead [1].

A meta-analysis including randomized trials, cohort studies and case-control studies failed to find a significant association between statin use and increased ICH [7]. They found a reduced ICH risk in statin users in case-control studies, but the relationship is not significant in cohort studies [7]. However, several meta-analyses only including randomized control studies did show a non-significant excess of hemorrhagic stroke in the groups of statin treatment [1, 36–38]. The similar trends were shown in the groups of randomized placebo-controlled trials of statin and ezetimibe [39, 40]. The ICH risk is not related to the degree of LDL-C reduction or achieved LDL-C [38]. However, an earlier meta-analysis, including 8832 patients with a history of cerebrovascular disease, found a significant increase in the risk of hemorrhagic stroke for statins users despite the reduced risk of ischemic stroke [41]. The level of LDL-C is no longer the treatment goal for hyperlipidemia in high risk patients in the current American College of Cardiology/American Heart Association task force guideline [33]. The effect of prior statin use in our study was consistent with other studies, neither associated with TC levels at presentation, nor with worse initial severity [42] and 3-month mortality in other studies [4, 43, 44]. Pre-ICH use of lipid-lowering drugs in small percentage of the ICH population (2.8%) was not associated with initial severity and 3-month outcomes, but prior use of statins were associated with better initial Glasgow Coma Scale score [4], and 3-month functional outcome in one study [9] but not in another [43].

Several studies have shown an increased ICH risk in extremely low or high BMI [45–47]. The present study demonstrated that the impact of low cholesterol on ICH severity and a worse prognosis was even more significant in patients with low BMI. BMI <22.0 kg/m^2^ was associated with 1.8-fold increase in the presenting ICH severity and nearly 4-fold increase in 3-month mortality. An eight-year hypertension follow-up study in China showed that either low or high BMI was associated with an increased risk in deep ICH, but not in lobar ICH [46]. A multi-center Italian case-control study found that obesity was associated with an increased risk of deep ICH indirectly through hypertension and diabetes mellitus, but without major effect on the risk of lobar ICH [48].

This study shows that a lower cholesterol level at the presentation of ICH is associated with worse initial severity and 3-months mortality, especially for those with a low BMI, but not with prior lipid-lower medication. A total cholesterol level lower than 160 mg/dL might be a concern in treating patients with dyslipidemia and high risk of intracerebral hemorrhage. For further verifying the conclusion, we performed further data analysis by establishing propensity score matched study groups and showed approximately similar findings. However, there are limitations to this study. First, the impact of high-density and low-density cholesterol was not investigated in the study. Second, cholesterol levels may vary overtime in the acute stage of stroke, although the difference was not significant in cerebral hemorrhage [49]. Third, the specific types of pre-ICH lipid-lowering drugs were not recorded in the registry and the percentage of pre-ICH lipid-lowering drug use was too small to have a solid conclusion of its impact on the ICH outcome. Fourth, fasting cholesterol levels were not measured in a large portion of ICH patients, and therefore the analysis might be weakened (S1 Table).

## Supporting information

S1 Table Characteristics of patients with intracerebral hemorrhage by total cholesterol levels on admission. Values are presented as mean ± standard deviation, number (percentage) or median (interquartile rang). TIA: transient ischemic attack, ICH: intracerebral hemorrhage; NIHSS: National Institute of Health and Stroke Scale. Chi-square test, ^†^ t- test and ^#^Wilcoxon rank-sum test.
(DOC)
