# Supplementary material for: Low cholesterol level associated with severity and outcome of spontaneous intracerebral hemorrhage: Results from Taiwan Stroke Registry
Source: PLoS One. 2017 Apr 19;12(4):e0171379. doi: 10.1371/journal.pone.0171379 (PMC5396870; doi:10.1371/journal.pone.0171379)
Supplement: S1 Table — Values are presented as mean ± standard deviation, number (percentage) or median (interquartile rang). TIA: transient ischemic attack, ICH: intracerebral hemorrhage; NIHSS: National Institute of Health and Stroke Scale. Chi-square test, † t- test and #Wilcoxon rank-sum test. (DOC) [file pone.0171379.s001.doc]

**Supporting information**

**S1 Table. Characteristics of Patients with Intracerebral Hemorrhage by Total Cholesterol Levels on Admission**

|  | All patients  n=6583 | Exclusion  n=4139 | Inclusion  n=2444 | *P* value |
| --- | --- | --- | --- | --- |
| Age, y | 62.1±14.9 | 61.9±15.3 | 62.5±14.2 | 0.09 |
| Male | 4274 (64.9) | 2704 (65.3) | 1570 (64.2) | 0.37 |
| Hypertension | 5546 (85.0) | 3444 (84.1) | 2102 (86.6) | 0.007 |
| Diabetes mellitus | 1635 (25.3) | 1016 (25.1) | 619 (25.6) | 0.66 |
| Previous ischemic stroke / TIA | 901 (13.8) | 567 (13.9) | 334 (13.7) | 0.88 |
| Previous ICH | 485 (7.51) | 298 (7.37) | 187 (7.75) | 0.57 |
| Atrial fibrillation | 312 (4.74) | 196 (4.74) | 116 (4.75) | 0.98 |
| Smoking | 2425 (37.4) | 1527 (37.6) | 898 (37.1) | 0.69 |
| Alcohol consumption | 1384 (21.3) | 859 (21.0) | 525 (21.6) | 0.57 |
| NIHSS score on admission | 13.0 (20.0) | 16.0 (21.0) | 9.00 (14.0) | <0.0001 |
| Surgery for ICH | 1759 (26.7) | 1403 (33.9) | 356 (14.6) | <0.0001 |
| Intraventricular hemorrhage | 1475 (22.4) | 1100 (26.6) | 375 (15.3) | <0.0001 |
| Stroke-in-evolution | 240 (3.7) | 148 (3.6) | 92 (3.8) | 0.69 |
| Infratentorial ICH | 473 (7.2) | 279 (6.7) | 194 (7.9) | 0.07 |
| Outcome at 3 months |  |  |  |  |
| Death | 1132 (17.2) | 932 (22.5) | 200 (8.2) | <0.0001 |
| Modified Rankin Scale >2 | 3578 (59.0) | 2496 (64.8) | 1082 (48.8) | <0.0001 |

Values are presented as mean ± standard deviation, number (percentage) or median (interquartile rang).

TIA: transient ischemic attack, ICH: intracerebral hemorrhage; NIHSS: National Institute of Health and Stroke Scale.

Chi-square test, ^†^ t- test and ^＃^Wilcoxon rank-sum test.
